# Phosphatase of Regenerating Liver-3 Expression Correlates With PTENP1/miR-21 and miR-17/PTEN Dysregulation in Endometrial Adenocarcinoma Progression

**DOI:** 10.14740/wjon2801

**Published:** 2026-07-30

**Authors:** Xin Xin Li, Yuan Xi Deng, Yu Xin Fu, Cai Xia Li, Ao Zhang, Jian Ming

**Affiliations:** a Department of Pathology, General Hospital of Northern Theater Command, Shenhe District, Shenyang 110016, Liaoning, China; b Dalian Medical University, Dalian, Liaoning, China; c Department of Pathology, Xi’an People’s Hospital (Xi’an No.4 Hospital), Xincheng District, Xi’an 710005, Shaanxi, China; d These authors contributed equally to this study.

**Keywords:** Endometrial adenocarcinoma, PRL-3, miR-21, miR-17, PTEN, PTENP1, HEC-1A cells

## Abstract

**Background:**

Endometrial cancer is a prevalent gynecologic malignancy, which is predominantly of the histological type known as endometrial adenocarcinoma. Deletion of the tumor suppressor gene *PTEN* contributes significantly to the development of endometrial cancer. Phosphatase of regenerating liver-3 (PRL-3), as a common pro-oncogenic factor, has been shown to promote angiogenesis in endometrial cancer. However, the functional relationship between PRL-3 and phosphatase and tensin homolog deleted on chromosome 10 (PTEN) in endometrial cancer remains unclear.

**Methods:**

Following the modulation of specific molecule expression, the levels of relevant molecules were assessed by quantitative real-time polymerase chain (qRT-PCR) and Western blot. Endometrial adenocarcinoma cell proliferation and migration were further determined by Cell Counting Kit-8 (CCK-8) assay and scratch assay.

**Results:**

PTEN and phosphatase and tensin homolog pseudogene 1 (PTENP1) expression in endometrial adenocarcinoma samples and cell lines was analyzed and found to be closely associated with the malignant biological behavior of tumors. High expressions of PTENP1 and PTEN inhibited the proliferative and migratory capacities of endometrial adenocarcinoma cells, and high expressions of PRL-3, miR-21 and miR-17 enhanced their proliferative and migratory capacities. Bioinformatics analysis predicted that PTEN and PTENP1 were direct targets of miR-21 and miR-17. PRL-3 could downregulate PTENP1 and PTEN through upregulation of miR-21 and miR-17, which in turn could enhance the proliferative and migratory capacities of endometrial adenocarcinoma.

**Conclusions:**

PRL-3 may play a pivotal role in promoting the proliferation and migration of endometrial adenocarcinoma by affecting the PTENP1/miR-21 or miR-17/PTEN.

## Introduction

Endometrial carcinoma (EC) ranks among the most prevalent gynecologic malignant tumors, which is the most commonly diagnosed gynecological cancer in developed countries [1]. The incidence of EC shows an increasing trend, and the prognosis is poorer for those diagnosed with advanced-stage disease [2]. The diagnosis of EC primarily relies on tissue sampling and invasive procedures, with surgery being the mainstay of treatment. Data from The Cancer Genome Atlas (TCGA) project have provided additional options for the management of EC [3, 4]. EC is predominantly composed of endometrial adenocarcinoma. Elucidating the mechanisms of EC initiation and progression is vital for advancing diagnostic approaches and targeted therapeutic strategies.

As a protein tyrosine phosphatase, Phosphatase of regenerating liver-3 (PRL-3) enhances the invasive and metastatic potential of tumor cells across a broad spectrum of cancers, with its overexpression serving as a marker of aggressive disease and poor outcomes. It modulates tumor cell behavior by regulating proliferation, migration, and metabolism [5], demonstrated in cancers such as metastatic colorectal cancer, glioma, and lymphoma [6–8]. Previous research has shown that PRL-3 upregulates vascular endothelial growth factor (VEGF) in endometrial adenocarcinoma, stimulating angiogenesis [9]. The potential mechanism through which PRL-3 exerts its oncogenic role in endometrial adenocarcinoma remains unclear.

Phosphatase and tensin homolog deleted on chromosome 10 (PTEN), as a tumor suppressor, exhibits both lipid phosphatase and protein phosphatase activities. It acts as an essential regulator to modulate malignant tumor cells progression, translation, apoptosis, and migration [10, 11]. The expression level of PTEN fluctuates throughout the menstrual cycle, while its high frequency of mutations in endometrial adenocarcinoma confirms its role as an early driving event in tumor progression [12, 13]. Multiple influencing factors can modulate the progression of endometrial adenocarcinoma by regulating PTEN expression. For instance, downregulation of miR-424 inhibits PTEN signaling, thereby suppressing tumor metastasis, while oleic acid exhibits inhibitory effects on tumor cell growth through the PTEN signaling pathway [14, 15]. PRL-3 can activate the phosphatidylinositol-3-kinase/protein kinase B (PI3K/AKT) pathway and suppress the expression level of PTEN, thereby promoting the malignant progression of hepatocellular carcinoma [16]. The regulatory mechanism between PRL-3 and PTEN has not been elucidated in endometrial adenocarcinoma.

Phosphatase and tensin homolog pseudogene 1 (*PTENP1*) is a processed pseudogene located at 9p13.3 [17]. *PTENP1* shares over 98% homology with PTEN in the coding region, and the 3' untranslated regions (3'UTRs) of both have been demonstrated to competitively bind microRNAs (miRNAs) [18, 19]. This hypothesis has been validated in cervical cancer, osteoporosis, and hepatocellular carcinoma, where PTENP1 competitively binds miRNAs to suppress PTEN expression [20–22]. The relationship among PTEN, PTENP1, and miRNAs in endometrial adenocarcinoma remains to be further studied.

miRNAs are key regulators of cell fate, proliferation, and death, and are frequently dysregulated in numerous cancers [23]. MiR-21 drives the development of many malignant tumors, including breast, ovarian, cervical, gastric, prostate, colorectal, and endometrial adenocarcinoma [24–27]. MiR-17 is highly expressed in bladder cancer [28]. Elevated miR-17 and miR-21 are observed in endometrial adenocarcinoma relative to adjacent normal samples [29, 30]. Therefore, we hypothesize that miR-17 and miR-21 serve as key regulators in the advancement and progression of endometrial adenocarcinoma, and that a potential competitive mechanism may exist between these miRNAs and PTENP1 to modulate PTEN expression. Preliminary experiments have shown that PRL-3 upregulates miR-21 and downregulates the level of both PTEN and PTENP1 in the endometrial adenocarcinoma cell line HEC-1A. However, the precise mechanism has yet to be fully elucidated.

This research aims to study the regulatory mechanisms of PRL-3 on miR-17, miR-21, PTEN, and PTENP1, and its impact on cellular proliferation and migration. Thereby providing a clearer understanding of the malignant biological behaviors of endometrioid adenocarcinoma and offering new insights into its pathogenesis and treatment.

## Materials and Methods

A total of 20 pairs of fresh endometrial adenocarcinoma samples along with matched adjacent non-cancerous tissues from patients were studied by our team at the General Hospital of Northern Theater Command between March and May 2023. All specimens were obtained from treatment-naive patients (without prior chemotherapy or other treatments) and were immediately stored at –80 °C until protein extraction. The human endometrial adenocarcinoma cell line (HEC-1A cells) was acquired from the Shanghai Institute of Cell Biology, CAS (Shanghai, China).

### Cell culturing and transfection

HEC-1A cells were cultured in McCoy’s 5A medium (Gibco, USA) supplemented with 10% fetal bovine serum (FBS; Gibco, USA), 100 U/mL penicillin, and 100 U/mL streptomycin at 37 °C in an incubator with 5% CO_2_ until they reached 75% confluence. The culture medium was then replaced with McCoy’s 5A medium containing active human recombinant PRL-3 (SRP0210; 100 ng/mL; Sigma) for further treatment. The overexpression plasmids, small interfering RNAs (siRNAs), and corresponding negative controls (NCs), including pcDNA3.1-PTEN, pcDNA3.1-PTENP1, PRL-3 siRNA, and PTEN siRNA, were obtained from Gene Chem (Shanghai, China). miR-17 mimic, miR-17 inhibitor, miR-21 mimic, miR-21 inhibitor, and miR-NC were purchased from KeyGEN Bio Tech (Nanjing, China).

One day before transfection, HEC-1A cells were seeded into six-well plates at a density of 5 × 10^5^ cells per well and cultured overnight to reach 70–90% confluence at the time of transfection. Lipofectamine™ 2000 (Invitrogen) was diluted in Opti-MEM™ reduced-serum medium and mixed gently. miRNA mimics, inhibitors, or corresponding negative controls were diluted separately in Opti-MEM™ to prepare individual master mixes. Each diluted transfection mix was then combined with the corresponding diluted Lipofectamine™ 2000, pipetted thoroughly, and incubated for 5 min at room temperature to allow complex formation. The resulting complexes were added dropwise to the cells and distributed evenly by gentle rocking of the plates. During the transfection period, cells were maintained in medium without antibiotics or serum to avoid potential interference with transfection efficiency. Six hours post-transfection, the medium was replaced with fresh complete growth medium containing 10% FBS and 1% penicillin–streptomycin. Transfected cells were incubated at 37 °C in a humidified atmosphere with 5% CO_2_ for 24–96 h before being harvested for subsequent assays.

Three siRNAs targeting PRL-3 were designed and synthesized by Nanjing KeyGen Biotech, with detailed information provided in Table 1. Transfection was performed at a final siRNA concentration of 50 nM. Then the siRNA sequence with the most pronounced and statistically significant knockdown effect was selected for subsequent experiments.

**Table 1:** The Sequence of PRL-3

Genes	Sequences
hPRL3si-1 sense	GGUGGAGGUGAGCUACAAATT
hPRL3si-1 antisense	UUUGUAGCUCACCUCCACCTT
hPRL3si-2 sense	CCUGUUCUCGGCACCUUAATT
hPRL3si-2 antisense	UUAAGGUGCCGAGAACAGGTT
hPRL3si-3 sense	CUACAAACACAUGCGCUUCCUTT
hPRL3si-3 antisense	AGGAAGCGCAUGUGUUUGUAGTT

miR-21 mimics, miR-21 inhibitor, miR-17 mimics, miR-17 inhibitor, and the corresponding negative controls were designed and synthesized by Nanjing KeyGen Biotech. Detailed information is provided in Table 2. The final transfection concentration was 50 nM for mimics and 100 nM for inhibitor.

**Table 2:** The Sequence of miRNA Mimics and miRNA Inhibitor

Genes	Sequences
hsa-miR-21 mimics sense	UAGCUUAUCAGACUGAUGUUGA
hsa-miR-21 mimics antisense	UCAACAUCAGUCUGAUAAGCUA
hsa-miR-21 inhibitor	UCAACAUCAGUCUGAUAAGCUA
hsa-miR-17 mimics sense	CAAAGUGCUUACAGUGCAGGUAG
hsa-miR-17 mimics antisense	CUACCUGCACUGUAAGCACUUUG
hsa-miR-17 inhibitor	CUACCUGCACUGUAAGCACUUUG
NC mimics sense	UUUGUACUACACAAAAGUACUG
NC mimics antisense	CAGUACUUUUGUGUAGUACAAA
NC inhibitor	CAGUACUUUUGUGUAGUACAAA

The PTEN and PTENP1 overexpression plasmids, along with the negative control plasmid, were constructed by GeneChem Biotech. And the vector backbone sequence was CMV enhancer–MCS–polyA–EF1α–zsGreen–sv40–puromycin. Detailed information is provided in Table 3.

**Table 3:** Plasmid Details

Genes	Species	Gene ID	GenBank	CDS size	Vector
*PTEN*	Human	5728	NM_000314	1,212	GV658
*PTENP1*	Human	11191	NR_023917	3,917	GV658

CDS: coding sequence.

### RNA extraction and quantitative real-time polymerase chain (qRT-PCR)

We extracted total RNA from the tissues and cells of endometrial adenocarcinoma using RNAiso Plus reagent (Takara, China). Cells were harvested by centrifugation at 1,000 rpm for 5 min at room temperature, washed once with ice-cold phosphate-buffered saline (PBS), and lysed in RNAiso Plus. After a 5-min incubation on ice, the lysate was mixed with 200 µL of chloroform, vigorously vortexed, and incubated on ice for 10 min. The mixture was then centrifuged at 13,000 rpm for 15 min at 4 °C. The aqueous phase (approximately 500 µL) was carefully transferred to a fresh RNase-free tube containing 500 µL of isopropanol, precipitated on ice for 10 min, and centrifuged at 13,000 rpm for 10 min at 4 °C. The resulting RNA pellet was washed once with 1 mL of 75% ethanol (12,000 rpm, 5 min, 4 °C), air-dried for 5–10 min, and dissolved in 20 µL of DEPC-treated water. RNA concentration and purity were assessed by spectrophotometry (A260/A280 ratio between 1.8 and 2.0).

Total RNA was reverse transcribed using the Prime Script RT Reagent Kit with gDNA Eraser from Dalian (Takara, China). Real-time PCR was conducted using the Applied Biosystems 7500/7500 Fast Real-Time PCR System (Thermo Fisher Scientific) with TB Green^®^ Premix Ex Taq™ II from Dalian (Takara, China). miRNA specific primers and U6 were synthesized by KeyGEN Bio Tech (Nanjing, China). U6 small nuclear RNA (snRNA) and β-actin were used as endogenous controls for miRNA and other genes, respectively. The qRT-PCR protocol consisted of an initial denaturation step at 95 °C for 30 s, followed by 40 cycles of denaturation at 95 °C for 5 s and annealing/extension at 60 °C for 34 s. The primer sequences used for PCR are provided in Table 4.

**Table 4:** Primer Sequences for qRT-PCR

Genes	Primer sequences
*PRL-3*	F: 5'-CTCATCACCCACAACC-3'
	R: 5'-CAGCCAGTCTTCCACTACCTT-3'
*PTEN*	F: 5'-TGGATTCGACTTAGACTTGACC-3'
	R: 5'-GGTGGGTTATGGTCTTCAAAAGG-3'
*PTENP1*	F: 5'-GAGTCGCCTGTCACCATTTC-3'
	R: 5'-TGTTGGAGGCAGTAGAAGGG-3'
*miR-21*	CTCAACTGGTGTCGTGGAGTCGGCAATTCAGTTGAGTCAACATC
	F: 5'-ACACTCCAGCTGGGTAGCTTATCAGACTGA-3'
	R: 5'-TGGTGTCGTGGAGTCG-3'
*miR-17*	GTCGTATCCAGTGCAGGGTCCGAGGTATTCGCACTGGATACGACCTACCT
	F: 5'-CCGCGCCAAAGTGCTTACAGTGC-3'
	R: 5'-ATCCAGTGCAGGGTCCGAGG-3'
*β-actin*	F: 5'-CTCGCCTTTGCCGATCC-3'
	R: 5'-GAATCCTTCTGACCCATGCC-3'
*U6*	F: 5'-CTCGCTTCGGCAGCACA-3'
	R: 5'-AACGCTTCACGAATTTGCGT-3'

qRT-PCR: quantitative real-time PCR.

### Western blot

Total protein was extracted using RIPA lysis buffer (Beyotime) supplemented with 1 mM phenylmethylsulfonyl fluoride (PMSF). All lysates were centrifuged at 12,000 rpm for 10 min at 4 °C, and the supernatants were collected as total protein extracts. Protein concentrations were determined using a bicinchoninic acid (BCA) assay kit (Beyotime). A standard curve was generated using serial dilutions of bovine serum albumin (BSA). All samples were tested in triplicate, and protein concentrations were calculated against the standard curve.

After electrophoresis on 10% or 12% sodium dodecyl sulfate (SDS)-polyacrylamide gel electrophoresis (PAGE) gels, the proteins were transferred onto PVDF membranes. The polyvinylidene difluoride (PVDF) membranes were incubated overnight at 4 °C with polyclonal rabbit anti-PRL-3 antibody (1:800; Abcam), monoclonal mouse anti-PTEN antibody (1:500; Santa), monoclonal mouse anti-glyceraldehyde-3-phosphate dehydrogenase (GAPDH) antibody (1:100000; Proteintech). Subsequently, the PVDF membranes were subjected to a 2-h incubation with the secondary antibody. Then, signals were detected using a chemiluminescence assays (Pierce, USA). Quantification of protein bands was performed with ImageJ software, with band intensities normalized to GAPDH and presented as relative integrated density values (IDVs).

### Cell proliferation assay

Transfected cells were seeded into 96-well plates at a density of 5,000 cells/well. We then added 10 µL of Cell Counting Kit-8 (CCK-8) solution (Absin Bioscience Inc., China) to each well, and the plates were incubated for 1–4 h. Absorbance at 450 nm was measured with a SpectraMax M5 enzyme labeler (Molecular Devices, USA). CCK-8 reagent was added at 24-h intervals, and absorbance at 450 nm was monitored until 96 h post-transfection.

### Wound-healing assay

We inoculated transfected or incubated HEC-1A cells into six-well plates and cultured to 90% cell volume after 24 h; artificial wounds were created with a sterile 200 µL lance tip. Following PBS washes, the suspended cells were resuspended and maintained in McCoy’s 5A medium with 2% serum at 37 °C. Cell morphology was then monitored at 0, 12, and 24 h using an inverted light microscope (Leica, Wetzlar, Germany) for photographic documentation. The migration rate (%) was determined by ImageJ software.

### Data analysis

To ensure the accuracy and reproducibility of the data, all experiments were repeated at least three times independently. All statistical analyses were performed using GraphPad Prism. Data are expressed as mean ± standard deviation (SD). Under the assumption of normal distribution, differences between experimental and control groups were examined using the *t*-test, with parametric tests applied when variances were equal and non-parametric tests when variances were unequal. For experiments involving two or more experimental groups, when the comparison was limited to differences between each experimental group and the control group, one-way analysis of variance (ANOVA) was used. A P value of < 0.05 was considered statistically significant (*P < 0.05, **P < 0.01, ***P < 0.001).

### Bioinformatic analysis

**Figure 1:**
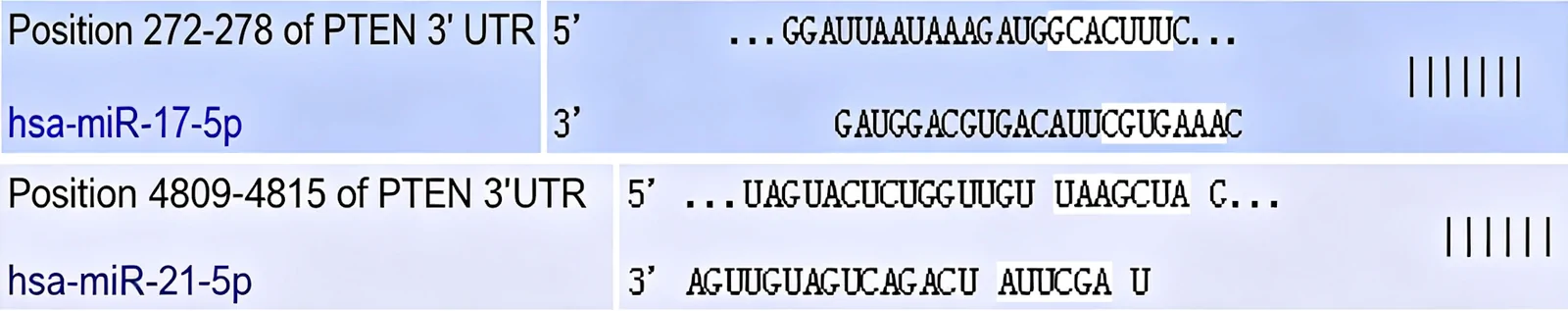
Relationship of miR-21 and miR-17 with PTENP1 and the binding site.

We employed the TargetScan bioinformatics suite to interrogate the predicted binding of miR-17/21 to PTEN and PTENP1(Fig. 1).

### Ethics approval

This study was approved by the Ethics Committee of the General Hospital of Northern Theater Command and was conducted in accordance with the Declaration of Helsinki.

## Results

### High PRL-3 expression and low PTEN expression in endometrial adenocarcinoma

**Figure 2:**
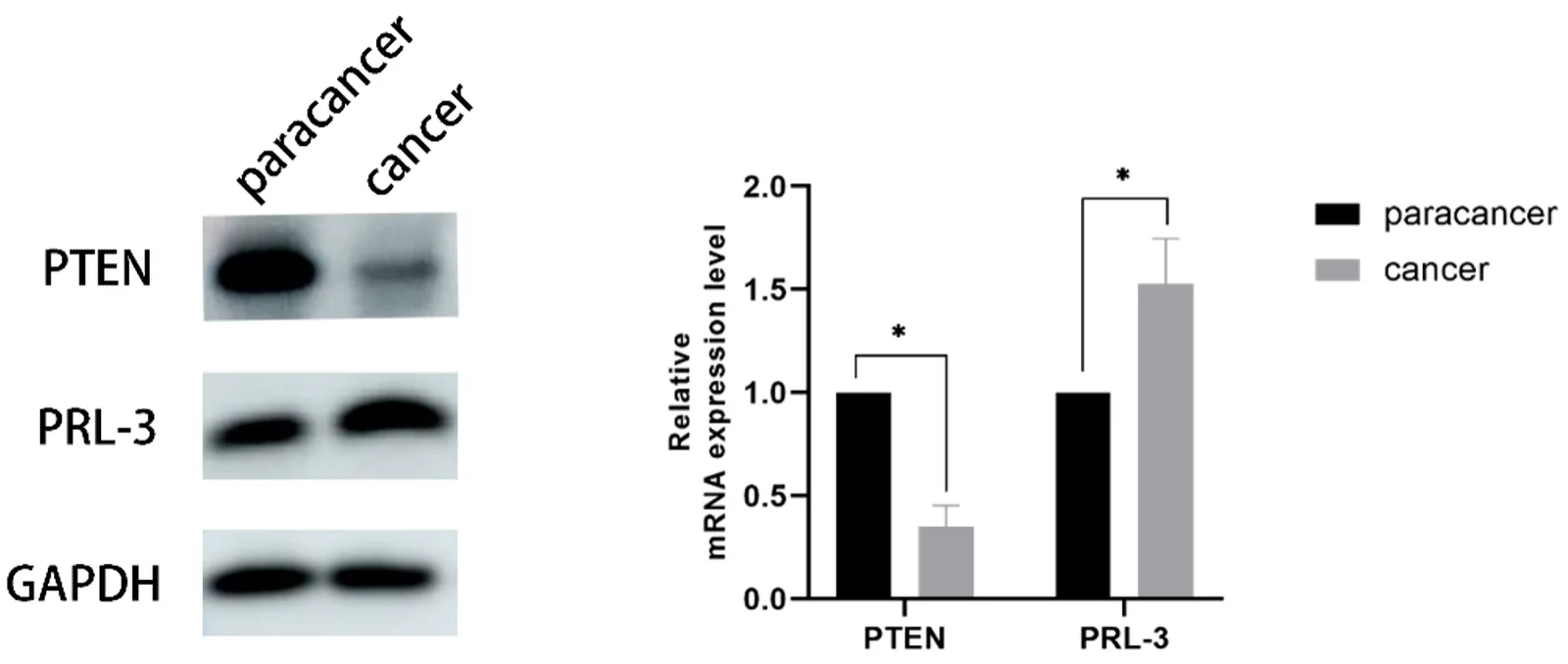
The protein expression of PTEN was decreased (P = 0.010) and the expression of PRL-3 was elevated (P = 0.030) in endometrial adenocarcinoma tissues. PTEN: phosphatase and tensin homolog deleted on chromosome 10; PRL-3: phosphatase of regenerating liver-3; GAPDH: glyceraldehyde-3-phosphate dehydrogenase.

The expression of PRL-3 in 20 pairs of endometrial adenocarcinoma tissues was detected by Western blot. Compared with the paracancerous tissues, the PTEN protein was markedly decreased, and the PRL-3 protein was elevated in endometrial adenocarcinoma (Fig. 2).

### PRL-3 promotes proliferation and migration of HEC-1A cells

**Figure 3:**
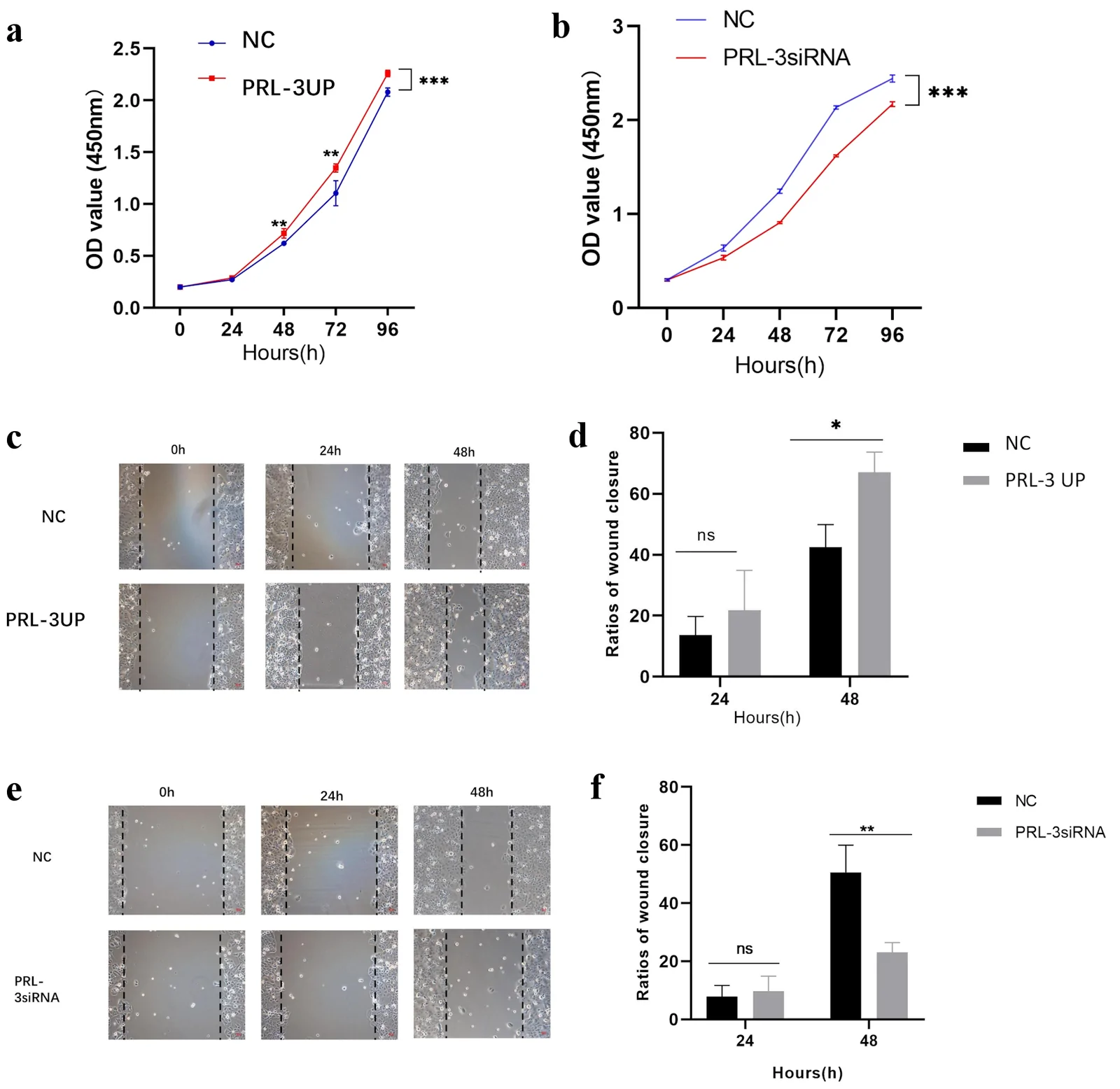
Effect of PRL-3 on the proliferation and migration of HEC-1A cells. (a) The OD value of HEC-1A cells in the PRL-3 overexpression group after 96 h of culture (P < 0.001). (b) The OD value of cells in the PRL-3 low-expression group after 96 h of culture (P < 0.001). (c, d) The migration rate of the scratched cells at 24 h in the PRL-3 overexpression group (P = 0.012). (e, f) The migration rate of scratched cells at 24 h in the PRL-3 low-expression group (P = 0.008). PRL-3: phosphatase of regenerating liver-3; NC: negative controls; OD: optical density; PRL-3 UP: PRL-3 upregulation.

To elucidate the regulatory role between PRL-3 and HEC-1A cells, we conducted CCK-8 and cell scratch assay in HEC-1A cells. The optical density (OD) value of HEC-1A cells in the PRL-3 overexpression group was significantly higher after 96 h of culture (Fig. 3a), and the migration rate of the scratched cells was significantly faster at 24 h (Fig. 3c, d). The OD value of cells in the PRL-3 low-expression group was significantly reduced after 96 h (Fig. 3b), and the migration rate of scratched cells was markedly slowed down at 24 h (Fig. 3e, f). Therefore, we speculated that PRL-3 enhances the proliferative and migratory capacities of HEC-1A cells.

### PRL-3 upregulates miR-21 and miR-17

**Figure 4:**
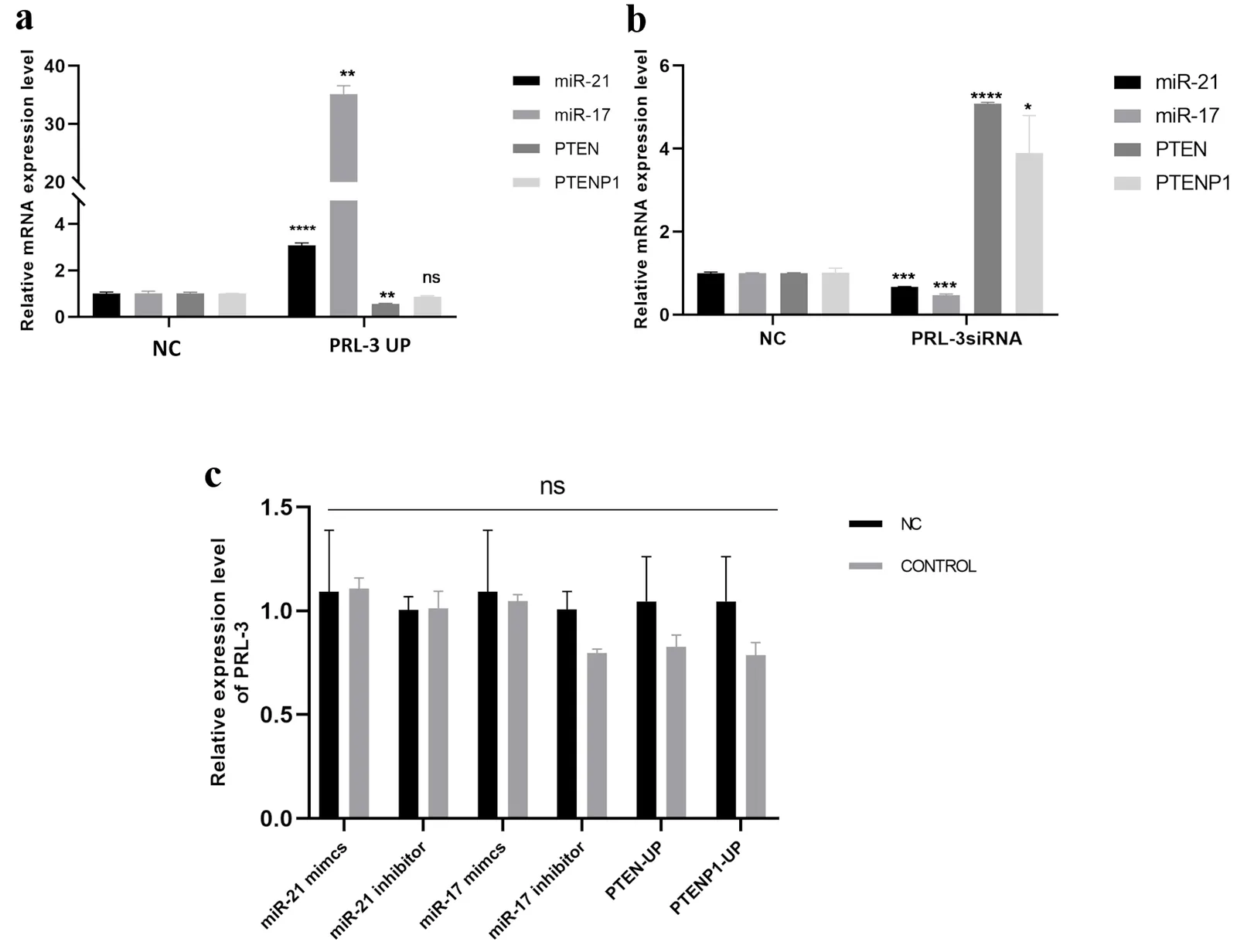
Effect of PRL-3 on the expression of miR-21 and miR-17. (a) miR-21/17 mRNA (P < 0.001, P = 0.001) and PTEN (P = 0.002)/PTENP1 mRNA expression in HEC-1A cells of the PRL-3 overexpression group. (b) miR-21/17 mRNA (P < 0.001, P < 0.001) and PTEN/PTENP1 mRNA (P < 0.001, P = 0.034) expression in HEC-1A cells of the PRL-3 low-expression group. (c) The changes of PRL-3 mRNA expression in response to both upregulation and downregulation of miR-21/17 and PTEN/PTENP1 (P > 0.05). PRL-3: phosphatase of regenerating liver-3; NC: negative controls; OD: optical density; PRL-3 UP: PRL-3 upregulation; PTENP1: phosphatase and tensin homolog pseudogene 1; PTEN: phosphatase and tensin homolog deleted on chromosome 10; PTENP1-UP: PTENP1 upregulation.

The result of qRT-PCR suggested that miR-21 and miR-17 mRNA were elevated in HEC-1A cells of the PRL-3 overexpression group (Fig. 4a). In PRL-3 low-expression group, miR-21 and miR-17 mRNA were decreased (Fig. 4b). And when miR-17 and miR-21 were upregulated or downregulated, no significant changes were observed in the level of PRL-3 mRNA (Fig. 4c). Therefore, we speculated that PRL-3 functions upstream to positively regulate miR-21 and miR-17 expression.

### PRL-3 downregulates PTEN and PTENP1

**Figure 5:**
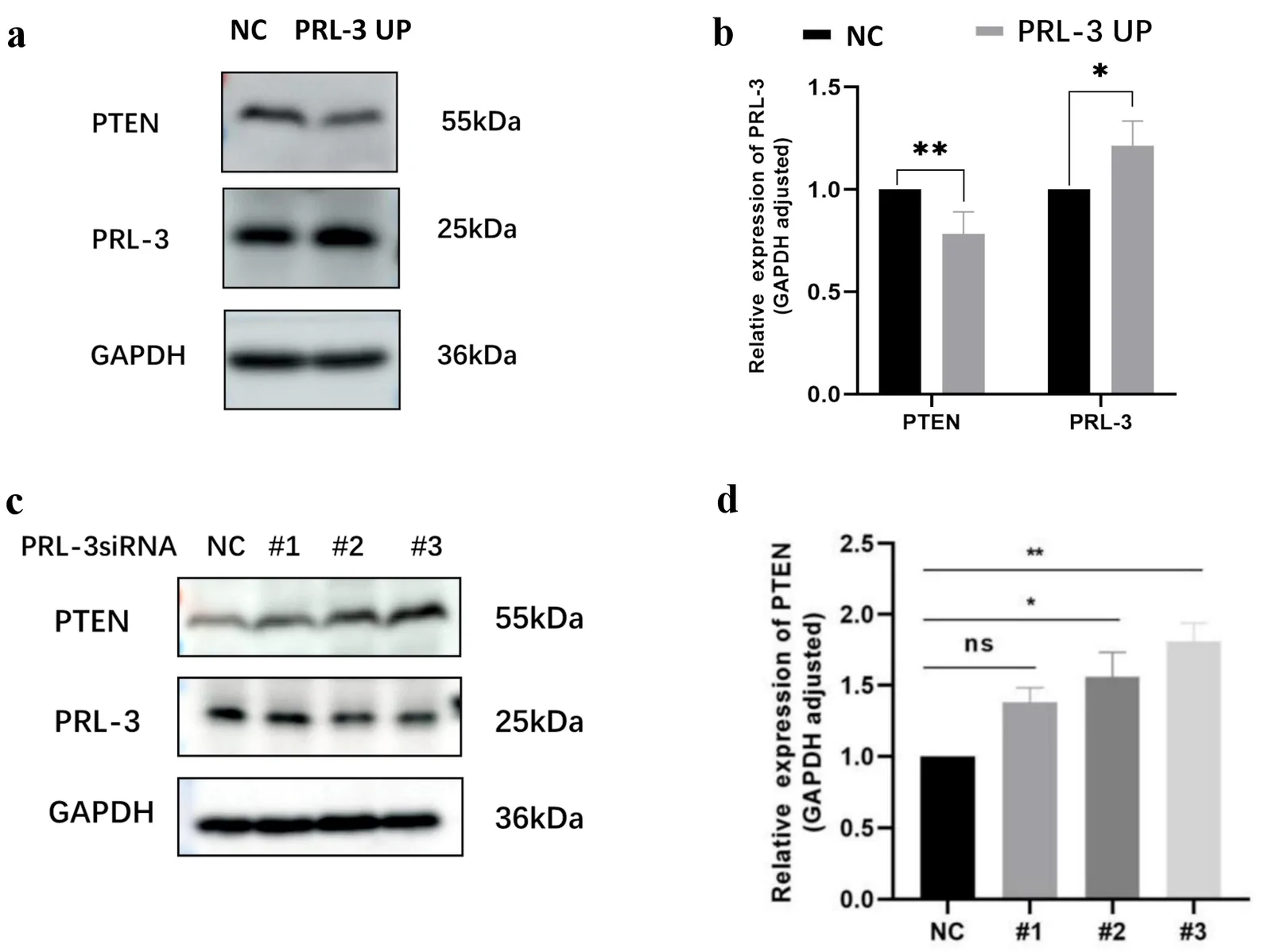
Effect of PRL-3 on the expression of PTEN and PTENP1. (a, b) The PTEN protein expression in HEC-1A cells of PRL-3 overexpression group (P = 0.009). (c, d) The PTEN protein in PRL-3siRNA-2 (P = 0.024) and PRL-3siRNA-3 (P = 0.003) HEC-1A cells of the PRL-3 low-expression group. PTEN: phosphatase and tensin homolog deleted on chromosome 10; PRL-3: phosphatase of regenerating liver-3; GAPDH: glyceraldehyde-3-phosphate dehydrogenase; ns: not significant; PRL-3 UP: PRL-3 upregulation; PTENP1: phosphatase and tensin homolog pseudogene 1; PTEN: phosphatase and tensin homolog deleted on chromosome 10.

The HEC-1A cells were divided into PRL-3 overexpression group and PRL-3 over low-expression group. QRT-PCR analyzed revealed that PTEN mRNA expression was reduced in HEC-1A cells in the PRL-3 overexpression group, in comparison with the control (Fig. 4a). PTEN and PTENP1 mRNA expression was increased in HEC-1A cells of PRL-3 low-expression group (Fig. 4b). No statistically significant changes in PRL-3 mRNA levels were detected following upregulation or downregulation of PTENP1 and PTEN (Fig. 4c). Western blot confirmed that PTEN protein was reduced in PRL-3 overexpression group of HEC-1A cells (Fig. 5a, b); PTEN protein was elevated in PRL-3siRNA-2 and PRL-3siRNA-3 HEC-1A cells of the group of PRL-3 low-expression (Fig. 5c, d). The qRT-PCR results were in agreement with the findings. Therefore, in endometrial adenocarcinoma tissues, PRL-3 could downregulate PTENP1 and PTEN, but PTEN and PTENP1 have no regulating effect on PRL-3.

### PTEN and PTENP1 inhibit proliferation and migration of HEC-1A cells

**Figure 6:**
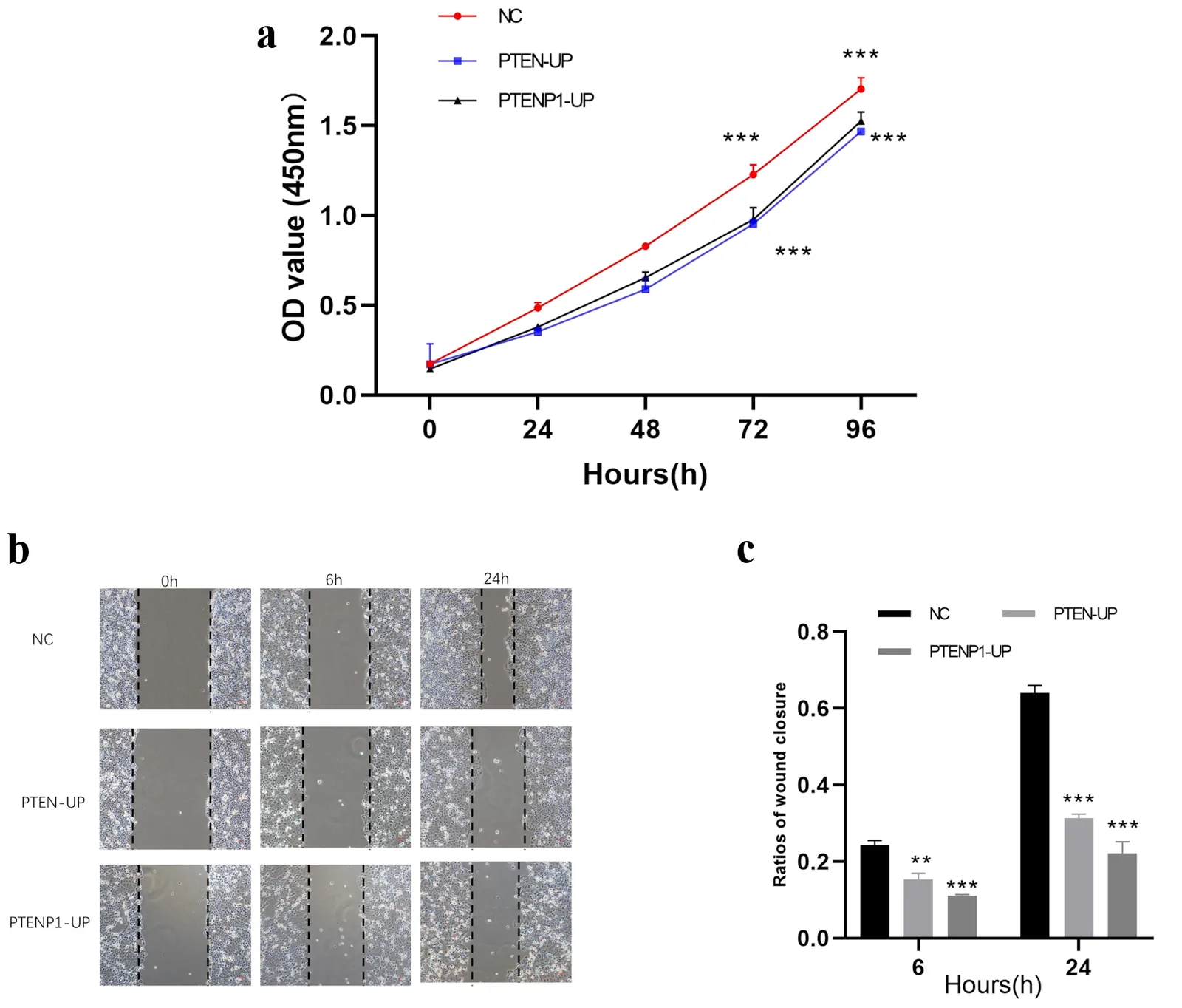
Effect of PTEN and PTENP1 on the proliferation and migration of HEC-1A cells. (a) The OD value of HEC-1A cells in PTEN and PTENP1 overexpression group after 96 h of culture (P < 0.001). (b, c) The migration rate of HEC-1A cells in PTEN and PTENP1 overexpression group at 24 h in the scratch assay (P < 0.001). PTENP1: phosphatase and tensin homolog pseudogene 1; PTEN: phosphatase and tensin homolog deleted on chromosome 10; OD: optical density; PTENP1-UP: PTENP1 upregulation.

The results of CCK-8 and cell scratch assay in HEC-1A cells suggested that the group of PTEN and PTENP1 overexpression exhibited a significantly reduced OD value in HEC-1A cells after 96 h of culture (Fig. 6a) and a significantly slower migration rate at 24 h in the scratch assay (Fig. 6b, c). In the occurrence and development of endometrial adenocarcinoma, PTEN and PTENP1 could suppress the proliferative and migratory capacities of HEC-1A cells.

### miR-21 and miR-17 promote the proliferative and migratory capacities of HEC-1A cells

**Figure 7:**
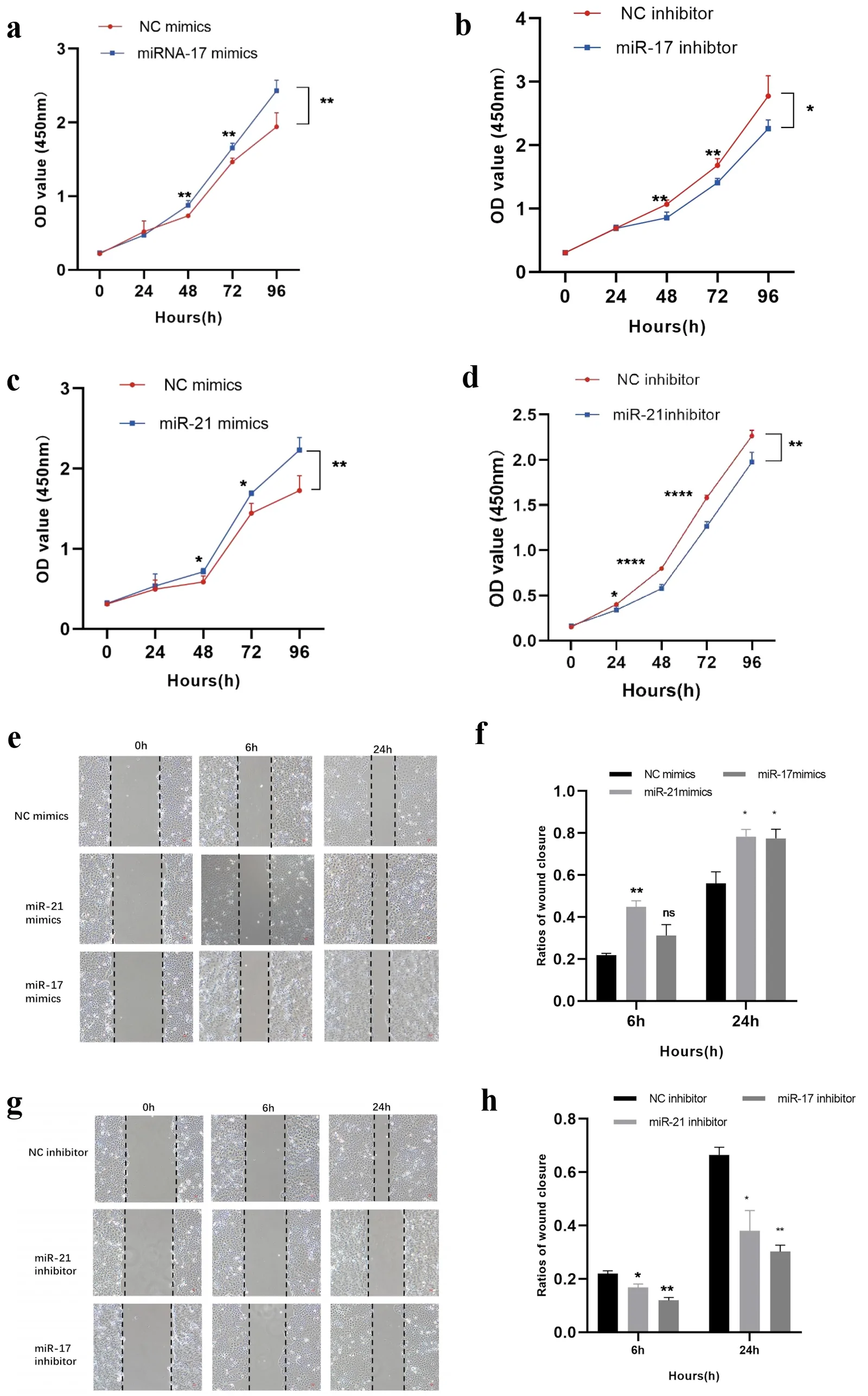
Effect of miR-21 and miR-17 on the proliferation and migration of HEC-1A cells. (a) The OD value of HEC-1A cells in miR-17 mimics group after 96 h of culture (P = 0.006). (b) The OD value of HEC-1A cells in the miR-17 inhibitor group after 96 h of culture (P = 0.025). (c) The OD value of HEC-1A cells in miR-21 mimics group after 96 h of culture (P = 0.006). (d) The OD value of HEC-1A cells in the miR-21 inhibitor group after 96 h of culture (P = 0.003). (e, f) The HEC-1A cells migration rate in miR-17 mimics group and miR-21 mimics group at 24 h (P = 0.026, P = 0.022). (g, h) The HEC-1A cells migration rate in the miR-17 inhibitor group and miR-21 inhibitor group at 24 h in the wound healing assay (P = 0.003, P = 0.011). OD: optical density; NC: negative controls.

The results of the CCK-8 and wound healing assays suggested that the mimics group of miR-17 and miR-21 exhibited a markedly higher OD value after 96 h of culture (Fig. 7a, c) and a markedly increased cell migration rate at 24 h (Fig. 7e, f). The HEC-1A cells in the inhibitor group of miR-17 and miR-21 exhibited a significantly lower OD value after 96 h of culture (Fig. 7b, d) and a significantly slower migration rate at 24 h in the wound healing assay (Fig. 7g, h). Therefore, miR-21 and miR-17 are critically involved in the development of endometrial adenocarcinoma, which promote growth and migration of endometrial adenocarcinoma cells.

### PTEN can upregulate PTENP1 and suppress miR-21/17, while PTENP1 can upregulate PTEN and inhibit miR-21/17

**Figure 8:**
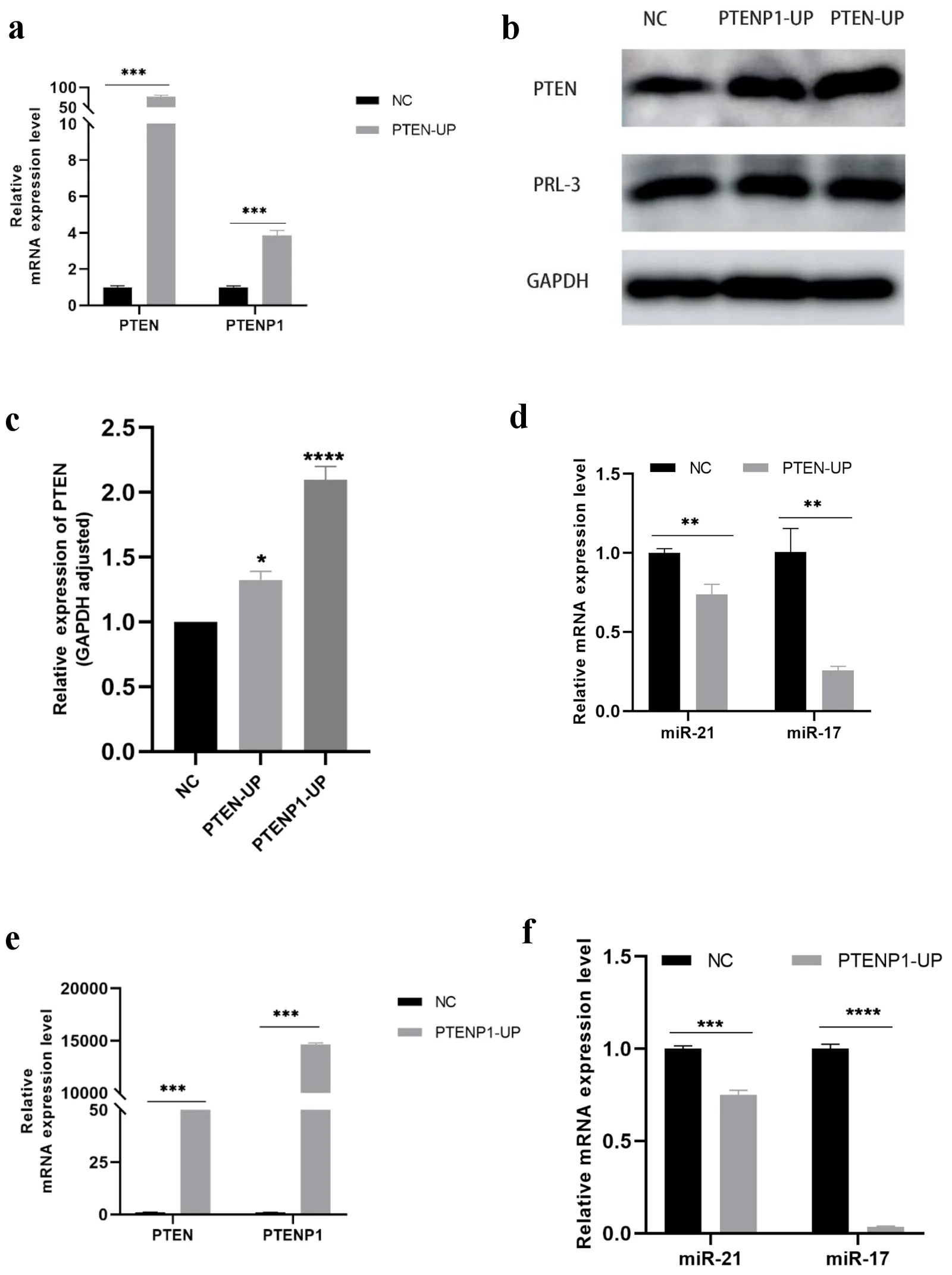
The regulatory interactions among PTEN, PTENP1, miR-21 and miR-17. (a) PTEN-overexpressing HEC-1A cells exhibited a significant increase in PTENP1 mRNA expression (P < 0.001). (b, c) PTEN protein expression was significantly higher in PTENP1-overexpressing HEC-1A cells (P < 0.001). (d) PTEN-overexpressing HEC-1A cells exhibited a significant decrease in miR-17 (P = 0.001) and miR-21 (P = 0.002) mRNA expression. (e) PTEN mRNA expression was significantly higher in PTENP1-overexpressing HEC-1A cells (P < 0.001). (f) The expression of miR-17 (P < 0.001) and miR-21 (P < 0.001) was markedly reduced in PTENP1-overexpressing HEC-1A cells. PTENP1: phosphatase and tensin homolog pseudogene 1; PTEN: phosphatase and tensin homolog deleted on chromosome 10; NC: negative controls; PTENP1-UP: PTENP1 upregulation.

To study the relation between miR-21/17 and PTEN/PTENP1, we constructed the groups of PTEN-overexpressing and PTENP1-overexpressing in HEC-1A cells. When PTEN was overexpressed, PTENP1 mRNA expression was significantly increased (Fig. 8a), alongside miR-17 and miR-21 mRNA were markedly decreased (Fig. 8d). PTEN mRNA expression was significantly higher in PTENP1-overexpressing HEC-1A cells (Fig. 8e), while miR-17 and miR-21 were markedly reduced (Fig. 8f). Meanwhile, the expression of PTEN protein was markedly increased in PTENP1-overexpressing HEC-1A cells (Fig. 8b, c). Therefore, PTEN and PTENP1 can mutually upregulate each other and both can suppress miR-17/21.

### miR-17 and miR-21 act as negative regulators of PTEN and PTENP1

**Figure 9:**
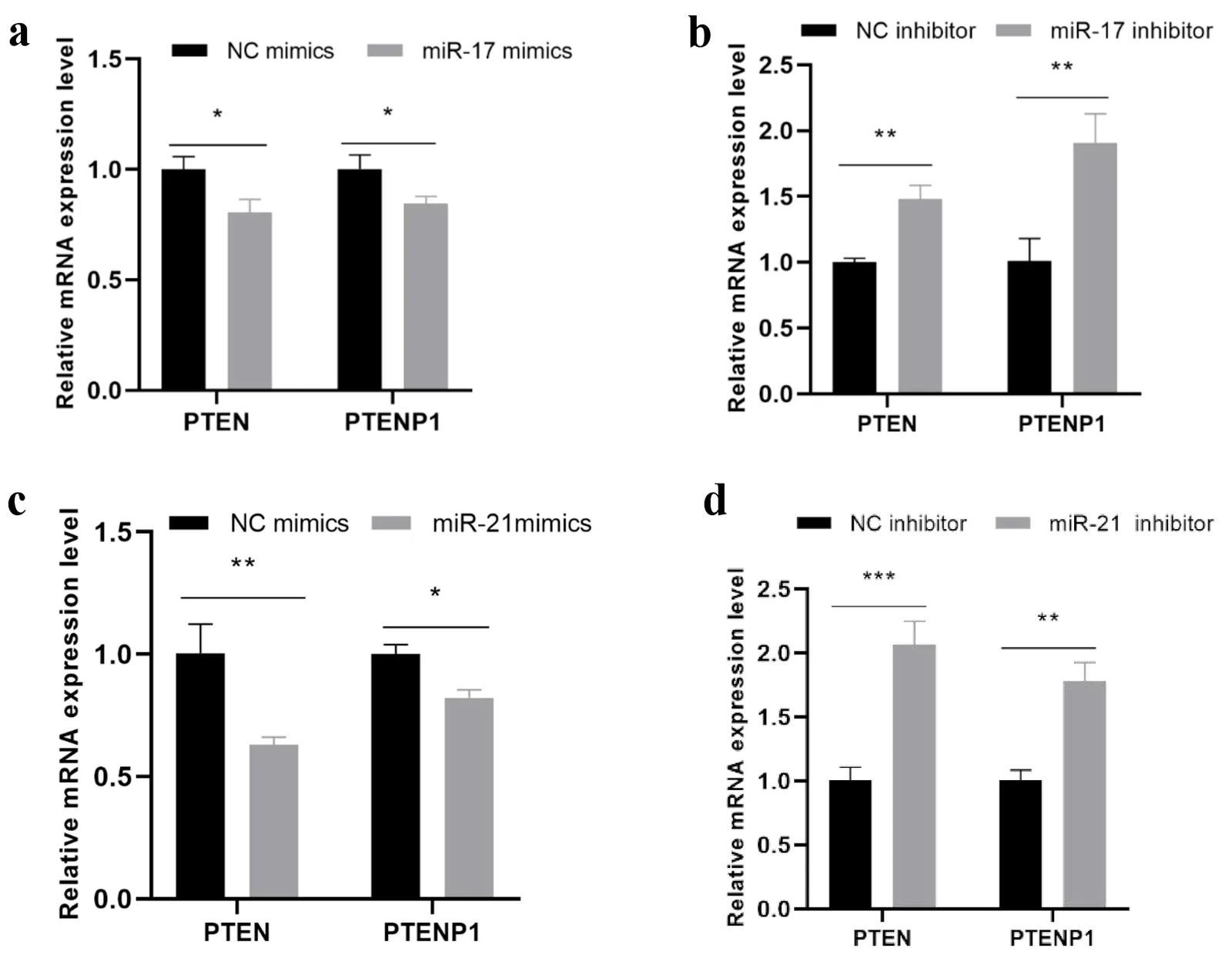
Effect of miR-17 and miR-21 on the expression of PTEN and PTENP1. (a) The miR-17 mimics group showed decreased expression of both PTEN mRNA (P = 0.014) and PTENP1 mRNA (P = 0.02) in HEC-1A cells. (b) The miR-17 inhibitor group exhibited increased expression of PTEN mRNA (P = 0.001) and PTENP1 mRNA (P = 0.005). (c) Transfection with miR-21 mimics led to reduced expression of PTEN mRNA (P = 0.005) and PTENP1 mRNA (P = 0.019). (d) The miR-21 inhibitor group demonstrated elevated expression of PTEN mRNA (P < 0.001) and PTENP1 mRNA (P = 0.001). PTENP1: phosphatase and tensin homolog pseudogene 1; PTEN: phosphatase and tensin homolog deleted on chromosome 10; NC: negative controls.

**Figure 10:**
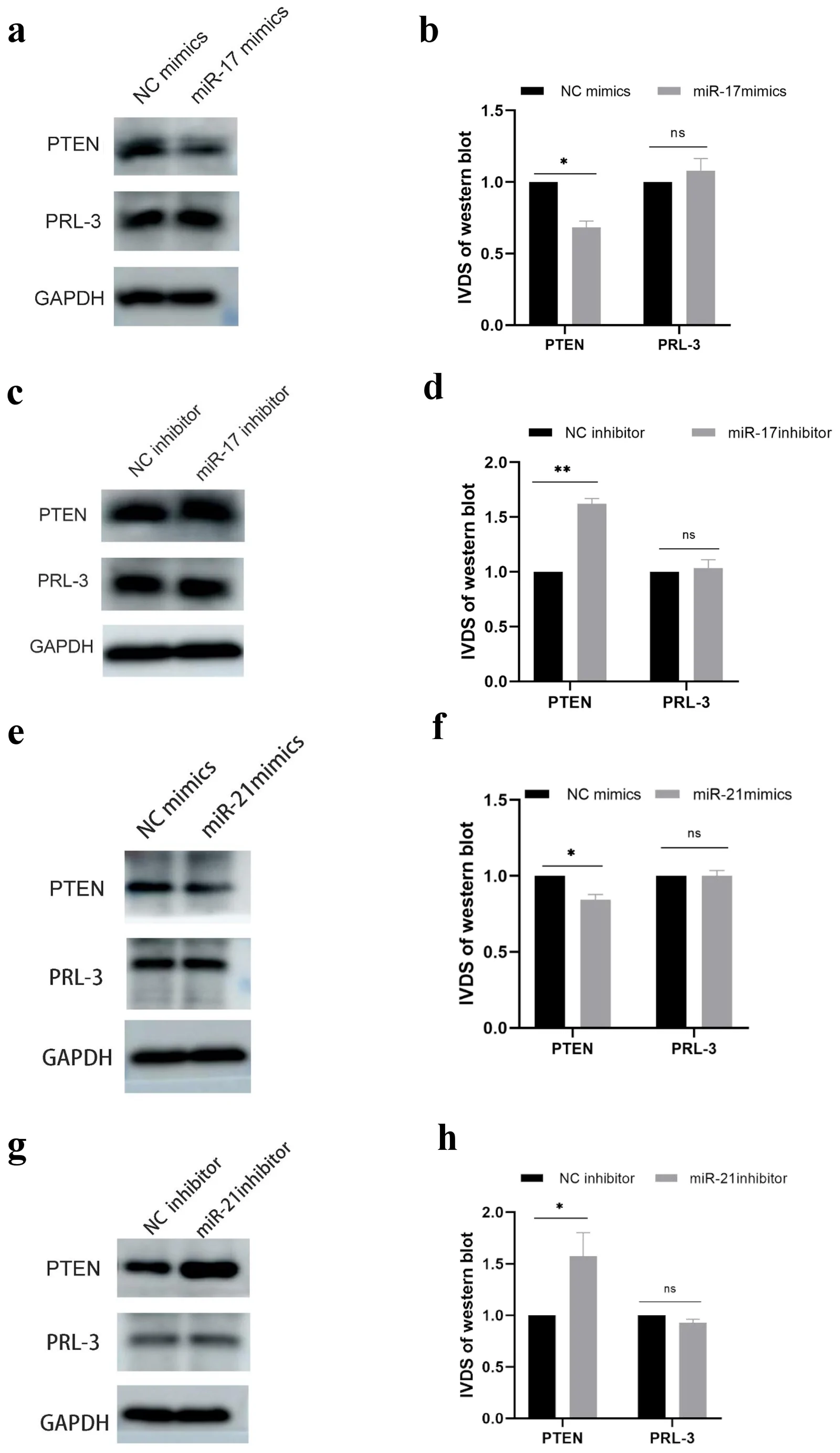
(a, b, e, f) The miR-17 mimics (P = 0.018) and miR-21 mimics (P = 0.045) groups exhibited decreased PTEN protein expression in HEC-1A cells. (c, d, g, h) The miR-17 inhibitor (P = 0.029) and miR-21 inhibitor (P = 0.006) groups showed increased PTEN protein expression. PTEN: phosphatase and tensin homolog deleted on chromosome 10; PRL-3: phosphatase of regenerating liver-3; GAPDH: glyceraldehyde-3-phosphate dehydrogenase; NC: negative controls.

The result of qRT-PCR revealed that the group of miR-17 mimics showed decreased expression of both PTEN mRNA and PTENP1 mRNA in HEC-1A cells (Fig. 9a); while the miR-17 inhibitor group exhibited elevated levels of PTEN mRNA and PTENP1 mRNA (Fig. 9b). Similarly, miR-21 mimics downregulated the mRNA level of PTEN and PTENP (Fig. 9c), whereas the miR-21 inhibitor group demonstrated elevated expression of PTEN mRNA and PTENP1 mRNA (Fig. 9d). The Western blot confirmed that both the miR-17 mimics (Fig. 10a, b) and miR-21 (Fig. 10e, f) mimics groups exhibited decreased PTEN protein expression in HEC-1A cells. Conversely, the miR-17 inhibitor (Fig. 10c, d) and miR-21 inhibitor groups (Fig. 10g, h) showed increased PTEN protein expression. Therefore, miR-17 and miR-21 mediate the downregulation of PTENP1 and PTEN.

### miR-17 and miR-21 counteract PTENP1-mediated inhibition of HEC-1A cells

**Figure 11:**
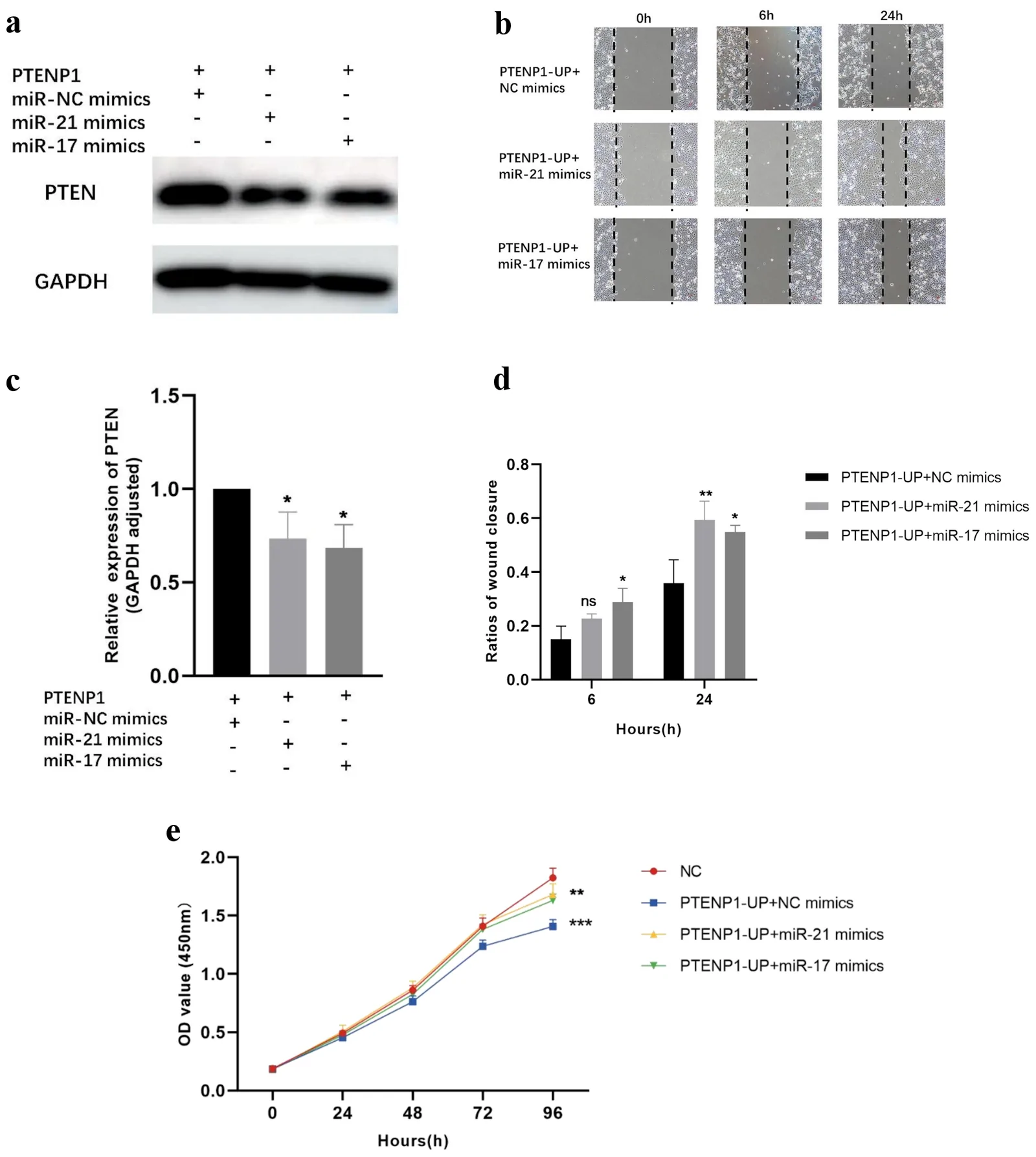
miR-17 and miR-21 reverse the inhibitory effect of PTENP1 on HEC-1A cells. (a, c) PTEN protein expression was decreased in HEC-1A cells of both the PTENP1-UP with miR-17 mimics group (P = 0.021) and the PTENP1-UP with miR-21 mimics group (P = 0.042). (b, d) The PTENP1-UP with miR-17 mimics (P = 0.021) and PTENP1-UP with miR-21 mimics (P = 0.008) groups exhibited a significantly increased cell migration rate at 24 h. (e) The PTENP1-UP with miR-17 mimics (P = 0.005) and PTENP1-UP with miR-21 mimics (P = 0.001) groups exhibited a significantly higher OD value after 96 h of culture. PTENP1-UP: PTENP1 upregulation; OD: optical density; PTEN: phosphatase and tensin homolog deleted on chromosome 10; GAPDH: glyceraldehyde-3-phosphate dehydrogenase; NC: negative controls.

The PTENP1 upregulation (PTENP1-UP) with NC mimics group served as the control group. Western blot analysis suggested that the PTEN protein level was decreased in HEC-1A cells of both PTENP1-UP with miR-17 mimics group (Fig. 11a, c) and PTENP1-UP with miR-21 mimics group (Fig. 11a, c). Results of the CCK-8 and wound healing assays demonstrated that both the PTENP1-UP with miR-17 mimics and PTENP1-UP with miR-21 mimics groups exhibited a significantly higher OD value after 96 h (Fig. 11e) and a markedly elevated cell migration rate at 24 h (Fig. 11b, d). The presence of miR-17 and miR-21 attenuates the inhibitory effect of PTENP1 on HEC-1A cells.

### PRL-3 enhances HEC-1A cell proliferation and migration through a miR-17/miR-21-mediated suppression of PTEN

**Figure 12:**
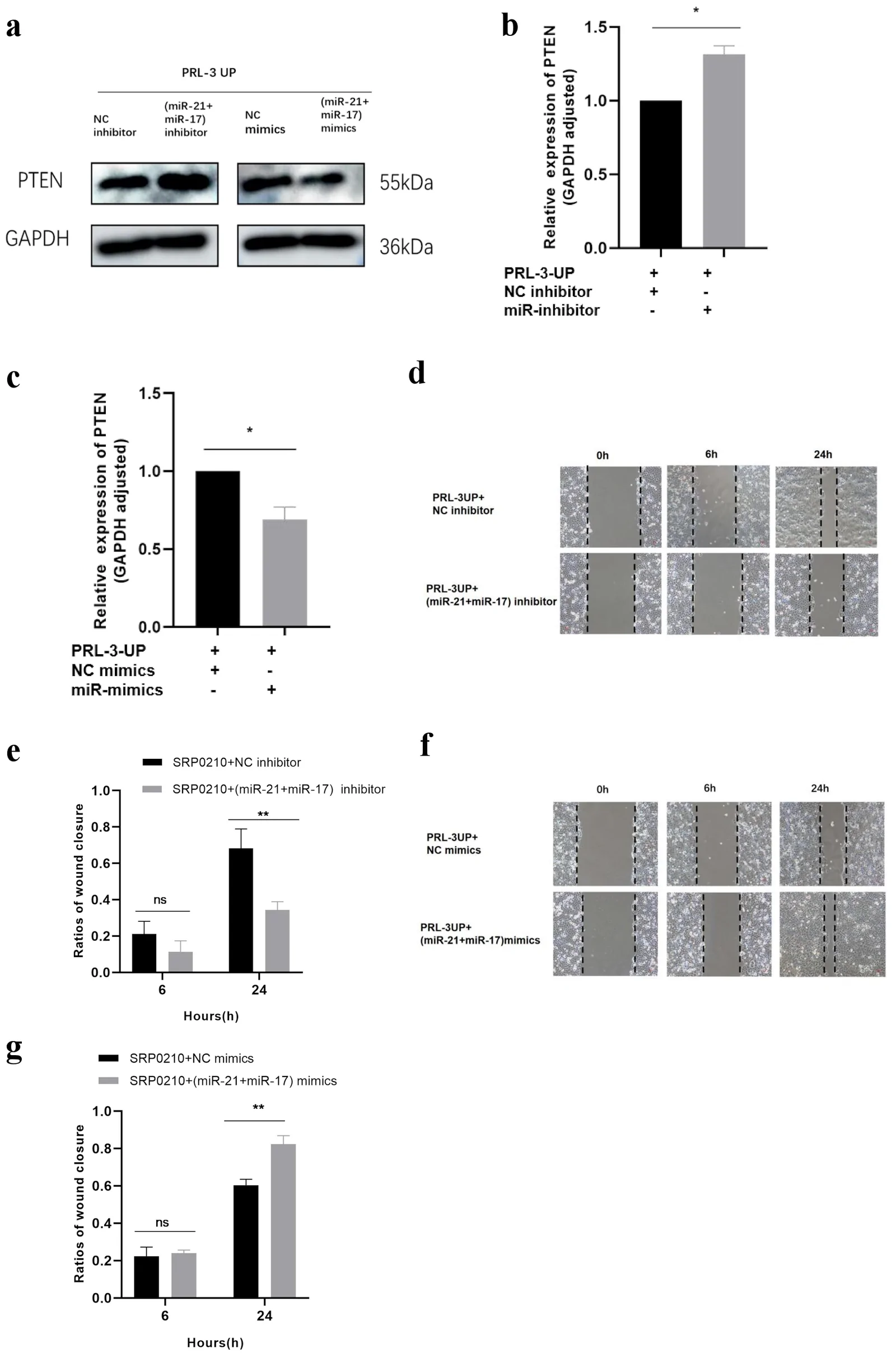
PRL-3 enhances HEC-1A cell proliferation and migration through miR-17/miR-21-mediated suppression of PTEN. (a–c) The PTEN protein expression of HEC-1A cells in PRL-3 overexpression with miR-17 and 21 inhibitors (P = 0.030)/mimics (P = 0.021) group and NC group. (d, e) The PRL-3 overexpression with miR-17/21 inhibitor group exhibited a significantly slower migration rate of HEC-1A cells at 24 h (P = 0.007). (f, g) The PRL-3 overexpression with miR-17/21 mimics group showed a significantly faster migration rate of HEC-1A cells at 24 h (P = 0.002). PTEN: phosphatase and tensin homolog deleted on chromosome 10; GAPDH: glyceraldehyde-3-phosphate dehydrogenase; NC: negative controls; PRL-3 UP: PRL-3 upregulation.

Western blot analysis revealed that the PRL-3 overexpression with miR-17 and miR-21 inhibitor group exhibited higher PTEN protein level in HEC-1A cells (Fig. 12a, b). In contrast, the PRL-3 overexpression with miR-17 and miR-21 mimics group showed reduced PTEN protein expression relative to the PRL-3 overexpression with NC mimics group (Fig. 12a, c). The wound healing assay confirmed that the PRL-3 overexpression with miR-17 and miR-21 inhibitor group exhibited a significantly slower migration rate of HEC-1A cells at 24 h (Fig. 12d, e). Conversely, the group of PRL-3 overexpression combined with miR-17 and miR-21 mimics showed a markedly faster migration rate relative to the PRL-3 overexpression with NC mimics group (Fig. 12f, g). Therefore, we speculated that PRL-3 enhances HEC-1A cell development through a miR-17/miR-21-mediated suppression of PTEN.

## Discussion

PRL-3 is participated in the metastasis of many malignancy tumors, which could promote the proliferative, migratory and invasive capacities of cancer. In our study, PRL-3 was highly expressed in the tissue of endometrial adenocarcinoma, while PTEN showed low expression levels. The overexpression of PRL-3 stimulates proliferation and migration in HEC-1A cells by upregulating miR-21/17 and downregulating PTEN/PTENP1. The opposite effects were observed when PRL-3 was downregulated. However, conversely, the level of PRL-3 was not affected by these molecules, including PTENP1, miR-17, miR-21, and PTEN.

PRL-3 mediates the aggressiveness of colorectal cancer through STAT3 activation, which transcriptionally upregulates miR-17 and miR-21 to enhance tumor growth and metastasis [31]. PRL-3 is highly expressed in endometrial adenocarcinoma and could promote the expression of VEGF by enhancing ERK phosphorylation. It shows a significant association with advanced clinical stage, metastases of lymph node, and poor survival [9]. Zhang et al [32] suggested that PRL-3 critically mediates the conversion of ovarian cancer cells into a cancer stem cell state. However, the regulating mechanism and underlying role of PRL-3 are remained uncleared in endometrial adenocarcinoma. In our study, we confirmed that PRL-3 can enhance the proliferative and migratory capacities of HEC-1A. PRL-3 acts upstream of PTENP1, miR-17, miR-21, and PTEN. Furthermore, PRL-3 facilitates HEC-1A cell aggressiveness by suppressing PTEN and PTENP1 through induction of miR-17 and miR-21 expression.

PTEN expression demonstrates a positive correlation with PTENP1 expression. High expression of PTENP1 and PTEN inhibits the proliferative and migratory ability of HEC-1A cells, whereas low levels promote these processes. Mutations and deletions in the *PTEN* gene represent a critical mechanism in endometrial adenocarcinoma tumorigenesis and progression, contributing to tumor proliferation, migration, and invasion [33]. As a pseudogene highly homologous to PTEN, PTENP1 is underexpressed in various cancer tissues. For example, downregulation of PTENP1 in cervical cancer tissues drives enhanced proliferation, migration, and invasion [20]. Additionally, downregulated PTENP1 expression in hepatocellular carcinoma, cervical cancer, and oral squamous cell carcinoma is associated with patient survival [26]. We confirmed that the roles of PTENP1 and PTEN in endometrial adenocarcinoma align with their profiles in other tumors. This groundwork lays a firm foundation for future research into the PTENP1- and PTEN-mediated signaling pathways in endometrial adenocarcinoma.

Abnormal expression of miRNAs occurs in cancer and diverse diseases, playing a role in the tumorigenesis and advancement of various malignant tumors. The miR-21 exhibits oncogenic properties via targeting multiple cancer suppressor genes, which consequently enhances tumor proliferation, invasive ability, and metastatic potential. The miR-17-92 cluster drives tumor advancement by targeting transforming growth factor (TGF)-β signaling components [34, 35]. This study demonstrates that miR-17 and miR-21 promote the malignant behaviors of HEC-1A cells. PTENP1 may act as a competing endogenous RNA (ceRNA) for miRNAs, upregulating the level of target genes and thereby regulating malignant biological behaviors in tumors.

Bao et al [36] revealed that PTENP1 upregulated PTEN by targeting miR-3611 to inhibit proliferative capacities of cervical cancer and promote EMT. Zheng et al [37] identified that PTENP1 serves as a molecular sponge for miR-17, thereby upregulating PTEN levels and influencing bladder cancer tumorigenesis. Zhang et al [38] identified that PTENP1 plays a role as a molecular sponge for miR-21, thereby upregulating PTEN expression in oral squamous cell carcinoma. The above studies demonstrated that PTENP1 regulates miRNAs through competitive binding to miRNAs, ultimately influencing tumor development. However, the expression level of miR-17 and miR-21 in endometrial adenocarcinoma and their mechanisms of action are not explicit. This study confirmed the negative relationship between miR-17/21 and PTENP1/PTEN: upregulation of miR-17/21 can inhibit PTEN and PTENP1, whereas downregulation of miR-17 and miR-21 produced the opposite effect.

When PTENP1 was elevated, miR-17 and miR-21 were decreased, and the PTEN was increased; when PTEN was elevated, miR-17 and miR-21 were decreased, and the expression of PTENP1 was increased. In other words, PTENP1 and PTEN can inhibit miR-17/21, and at the same time, PTENP1 and PTEN positively regulate each other. The positive regulatory role of PTEN by PTENP1 was reversed when PTENP1 and miR-17 and miR-21 were simultaneously upregulated compared to only PTENP1. This phenomenon was also verified in cell proliferation and scratch assays. Compared with the upregulation of PTENP1 only, the proliferative and migratory capacities of HEC-1A cells were accelerated when the level of PTENP1, miR-17 and miR-21 were simultaneously upregulated. Therefore, miR-17 and miR-21 antagonize the cancer-suppressive role of PTENP1 in HEC-1A cells, and the suppression mediated by PTENP1 on cells was dependent on the regulation of PTEN. PTENP1 functions as a “molecular sponge” to compete with miR-17 and miR-21 while restoring PTEN function in endometrial adenocarcinoma. PTENP1-miR-17/21-PTEN together form a ceRNA network that plays a key role in endometrial adenocarcinoma advancement.

Therefore, in endometrial adenocarcinoma tissues, PRL-3 was elevated while PTEN was reduced, compared with paracancerous tissues. Therefore, we predict *PTEN* and *PTENP1* may both are target genes of miR-17 and miR-21, which competitively bind these miRNAs. PTEN expression is positively regulated by PTENP1, while the regulation of PTEN by PTENP1 may depend on miR-17 and miR-21. PRL-3 is located in the upstream of ceRNA regulatory mechanism, which upregulates miR-17 and miR-21 to downregulate PTEN and PTENP1. However, how PRL-3 mediates the upregulation of miR-17/21 and affects the downstream pathways still needs to be further investigated. Verification of PTEN, PTENP1 and miR-17/21 target binding sites is also a major direction for future research.

Several limitations of this study should be acknowledged. First, our findings were obtained solely from HEC-1A cells, and whether they extend to other endometrial cancer cell lines remains to be determined. Second, the miR-17/miR-21 and PTEN/PTENP1 interactions were predicted and require direct experimental verification using dual-luciferase reporter assays. These two regulatory axes have been partially supported by previous studies in endometrial cancer [39, 40], and our results are consistent with these observations. Future studies incorporating additional cell lines and direct binding assays will be necessary to further validate the conclusions.

### Conclusions

Endometrial adenocarcinoma tissues showed high expression level of PRL-3 and low expression level of PTEN compared with paracancerous tissues. *In vitro* studies, PRL-3, miR-17 and miR-21 can enhance the proliferative and migratory ability of HEC-1A cells, and PTENP1 and PTEN inhibited the proliferative and migratory capacities of HEC-1A cells. Therefore, PRL-3 may promote HEC-1A cell aggressiveness through dysregulation of the PTENP1-miR-17/21-PTEN network, although the precise regulatory mechanisms remain to be elucidated.

## Data Availability

The data that support the findings of the present study are available from the corresponding author on reasonable request.

## Abbreviations

PRL-3 UP: PRL-3 upregulation
PTENP1-UP: PTENP1 upregulation
PTENP1: phosphatase and tensin homolog pseudogene 1
PTEN: phosphatase and tensin homolog deleted on chromosome 10
PRL-3: phosphatase of regenerating liver-3
qRT-PCR: quantitative real-time PCR
CCK8: Cell Counting Kit-8
TCGA: The Cancer Genome Atlas
VEGF: vascular endothelial growth factor
miRNA: microRNA
NC: negative controls
ceRNA: competing endogenous RNA
SRP0210: recombinant human PRL-3
ns: not significant
